# Surgical treatment of intramyocardial dissecting hematoma—a case report and literature review

**DOI:** 10.3389/fcvm.2025.1700770

**Published:** 2025-12-01

**Authors:** Mark Mervic, Andreja Cerne Cercek, Ana Ovsenik, Juš Kšela, Luka Lipar

**Affiliations:** 1 Faculty of Medicine, University of Ljubljana, Ljubljana, Slovenia; 2 Clinical Department of Cardiology, Internal Medicine Clinic, University Medical Centre Ljubljana, Ljubljana, Slovenia; 3 Clinical Department of Cardiovascular Surgery, Surgical Clinic, University Medical Centre Ljubljana, Ljubljana, Slovenia

**Keywords:** intramyocardial dissecting hematoma, myocardial infarction, cardiac imaging, surgical repair, case report

## Abstract

**Background:**

Intramyocardial dissecting hematoma (IDH) is a rare but potentially life-threatening complication, most commonly linked to myocardial infarction (MI) but also described in other settings such as cardiac interventions or severe chest trauma. Owing to the limited data on its diagnosis, management, and outcomes, treatment strategies for IDH remain controversial.

**Case presentation:**

We present a case of a 53-year-old female who was initially misdiagnosed with Takotsubo cardiomyopathy and later found to have an IDH complicated by anticoagulation dilemmas and infection. Despite initial conservative management, progressive dissection and elevation of inflammatory markers prompted surgical intervention. The patient underwent endoventricular patch repair (Dor procedure), with successful resolution and functional recovery through rehabilitation.

**Conclusions:**

This case highlights the importance of keeping IDH in the differential diagnosis, especially in patients with non obstructive coronary findings or unexplained myocardial wall abnormalities. Multimodal imaging, especially cardiac MRI, plays a key role in confirming the diagnosis and determining the extent of dissection. Treatment should be highly individualized, balancing the risks of hematoma expansion against the need for anticoagulation when intracavitary thrombi are present. Infection risk can further complicate the clinical course, necessitating early recognition and intervention. When conservative management is no longer viable, surgical repair—such as the Dor procedure—can stabilize the myocardium and lead to meaningful recovery.

## Background

Intramyocardial dissecting hematoma (IDH) is an exceedingly rare potentially life-threatening mechanical complication of myocardial infarction (MI), characterized by a blood-filled cavity within the necrotic myocardial layers (1, 2). It has been described as a partial or subacute cardiac rupture, with the dissecting plane contained between the epicardium and the endocardium, and it can rapidly progress to a complete wall rupture if not promptly recognized (2, 3). Although IDH most often appears in the free wall of the left ventricle (LV), it may also arise in the interventricular septum or the right ventricular wall (4). In addition to myocardial infarction, IDH may also occur following percutaneous coronary interventions (PCI), cardiac surgery, severe chest trauma, or spontaneously, further underscoring its heterogeneous etiologies (5, 6).

Transthoracic echocardiography (TTE) frequently serves as the first-line imaging modality for IDH, detecting new cavitation or echo-lucent spaces within the myocardium. Nonetheless, it may be challenging to distinguish IDH from other entities such as LV thrombus or pseudoaneurysm (5, 7). Contrast-enhanced echocardiography can facilitate this differentiation by outlining endocardial borders (1), while cardiac magnetic resonance imaging (MRI) is often regarded as the gold standard due to its superior tissue characterization and ability to confirm the presence of hemorrhage within the myocardial wall (4, 8).

Despite increased awareness and better diagnostic tools, the clinical management of IDH remains controversial (5). Some patients with stable hemodynamics and contained hematomas can be managed conservatively, with cases of spontaneous regression documented in the literature (9, 10). In contrast, a subset of individuals—especially those exhibiting signs of progressive dissection, hemodynamic instability, or ventricular septal defect-require urgent surgical intervention (4, 8).

Given the pronounced heterogeneity and the absence of large clinical trials or definitive guidelines, decision-making frequently hinges on a tailored, multidisciplinary approach involving cardiologists, cardiac surgeons, and imaging specialists (7, 11).

We present a case of a 53-year-old female initially presumed to have Takotsubo cardiomyopathy who was ultimately diagnosed with an IDH complicated by infection and anticoagulation dilemmas, culminating in surgical repair.

## Case presentation

A 53-year-old female patient, a long time smoker (30 pack-years), with a history of hepatic steatosis and recent (3 months prior) use of rivaroxaban (Xarelto) for suspected yet unconfirmed deep vein thrombosis (DVT), presented to a regional hospital due to sudden, pressure-like retrosternal chest pain lasting about 3 h, accompanied by mild dyspnea, without diaphoresis, dizziness or syncope. The pain, previously unknown to her, subsided with nitroglycerin. An initial electrocardiogram (ECG) demonstrated T-wave inversions in leads II, III, aVF, and ST segment elevations in V3-V6 (Supplementary Figure S1), prompting a transfer to a tertiary centre for suspected acute anterior wall ST-elevation myocardial infarction (STEMI).

### Initial investigations and diagnostic dilemma

Laboratory investigations revealed moderately elevated high-sensitivity troponin I (687 ng/L), and N-terminal pro-B-type natriuretic peptide (NT-proBNP) (2,247.7 ng/L). Urgent coronary angiography showed no significant coronary artery stenosis (Supplementary Figure S2). However, left ventriculography showed apical dyskinesia, suggestive of Takotsubo cardiomyopathy. She was managed conservatively and monitored in the intensive care unit, remained hemodynamically stable, afebrile, and did not require supplemental oxygen. Serial cardiac biomarkers showed a declining trend, with troponin levels decreasing to 280 ng/L in the next two days.

**Figure 1:**
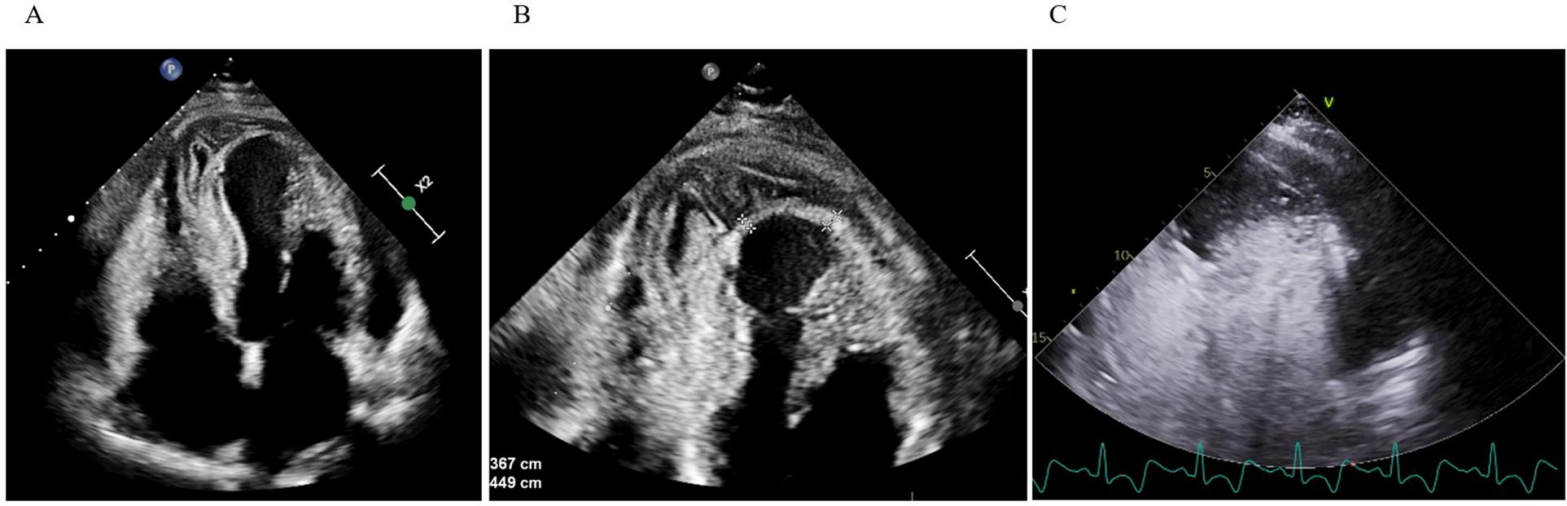
Transthoracic echocardiography. **(A,B)** Apical four-chamber view shows apical and inferoseptal myocardial thickening with a large, echolucent, spindle-shaped intramyocardial cavity, without evident communication to cardiac chambers or pericardial space. **(C)** Contrast echocardiography demonstrates no flow from the ventricular cavity into the lesion.

Initially diagnosed as stress-induced myocardial dysfunction, she was transferred back to a secondary hospital for further observation. Shortly thereafter, she became febrile (temperature up to 38.2°C) and was treated with oral penicillin for streptococcal pharyngitis. Despite therapy, inflammatory markers (CRP 192 mg/L) remained high, and troponin rose to 4,584 ng/L. TTE on September 16 demonstrated a moderately dilated LV [end-diastolic diameter (EDD) 60 mm], dyskinesia of the apical segments of the LV, mild pericardial effusion, and suspicious “layering” in the LV wall consistent with intramyocardial bleed or pseudoaneurysm (Figure 1).

Subsequent thoracic computed tomography (CT) confirmed an extensive transmural infarction involving the inferoapical LV with signs of hemorrhage into the necrotic myocardial wall and moderate pericardial effusion, excluding transient Takotsubo pattern and suggesting a complicated ischemic event. Rivaroxaban was discontinued, and heart failure treatment (spironolactone 25 mg OD, valsartan and sacubitril 24/26 mg BiD, bisoprolol 2,5 mg OD) was initiated.

Given the concern for intramyocardial extension of the infarction, she was transferred back to the tertiary centre's cardiology department on day 5 for advanced evaluation.

### Progressive intramyocardial hematoma

**Figure 2:**
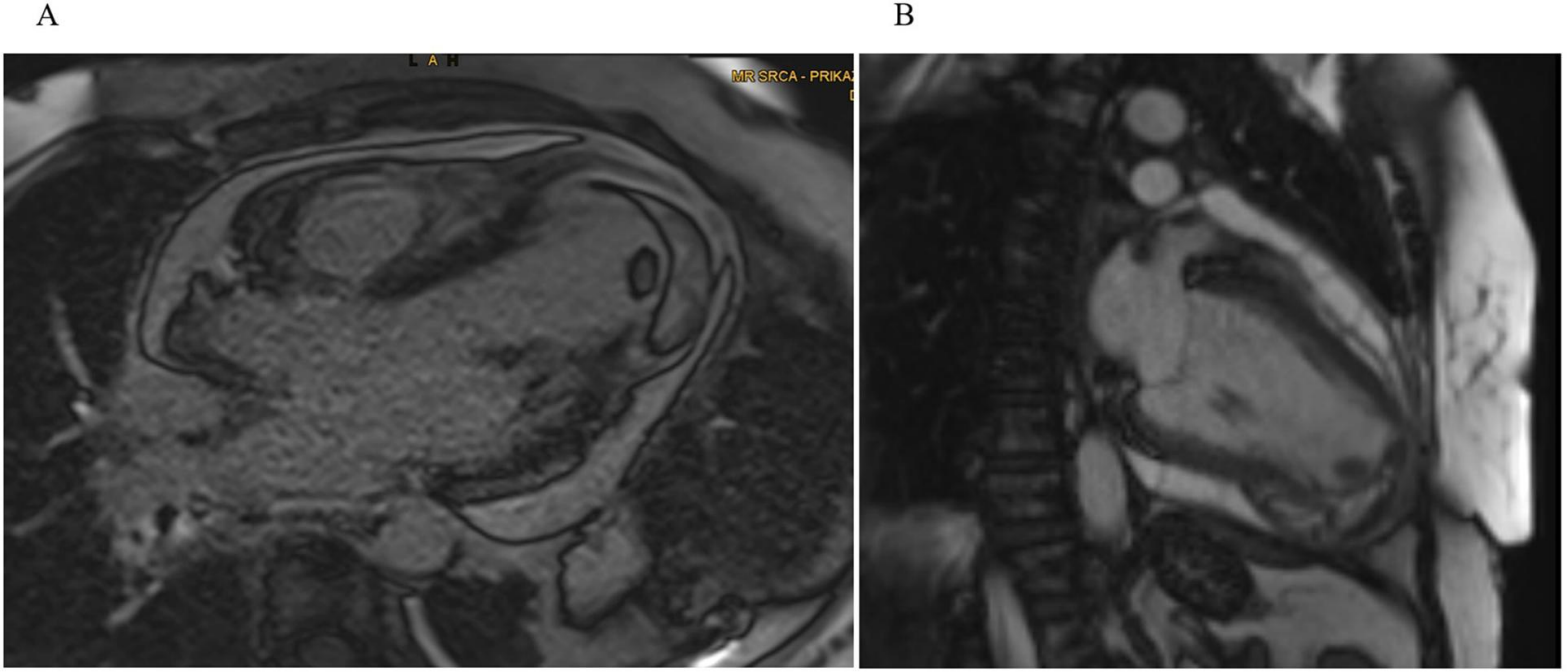
Cardiac MRI. **(A)** Four-chamber and **(B)** two-chamber views showing a well-demarcated hypointense lesion within the inferoapical wall extending into the septum, consistent with intramyocardial hemorrhage and a clearly defined intracavitary thrombus visible at the LV apex.

Upon admission back to the tertiary cardiac care unit (CCU), cardiac MRI on day 6 confirmed an inferoapical transmural MI extending into the septum, complicated by a large IDH (60 × 55 × 30 mm). An apical intracavitary thrombus measuring 10 mm was also identified. Findings of acute pericarditis (extensive inflammation and late gadolinium enhancement) and pericardial effusion (up to 15 mm) along the lateral wall of LV (Figure 2). Concurrently, aspirin at a dose of 100 mg was introduced for antiplatelet effects because of the suspicion of myocardial infarction as the primary event and colchicine was added for pericarditis management.

Balancing the risk of hematoma expansion vs. the risk of systemic embolization due to the LV apical thrombus, a low-intensity anticoagulation approach with continuous unfractionated heparin infusion was started allowing immediate reversal if needed.

Over the following weeks, TTE imaging revealed a mild increase in hematoma size to 62 × 44 × 32 mm associated with worsening apical dyskinesia and persistent intracavitary thrombus, now measuring 20 × 12 × 12 mm, despite anticoagulation (Supplementary Figure S3). During this time Troponin I levels slowly declined (94 → 23 ng/L by day 12) while CRP initially remained elevated (210 mg/L on day 10, fluctuating from about 120 to 190 mg/L in the following days).

Given the complex interplay of hematoma, thrombus, and possible myocardial rupture a multidisciplinary meeting involving cardiologists, cardiac surgeons, and anticoagulation specialists was organized on day 21. It was agreed that the patient was at higher immediate risk from the expanding thrombus than from the hematoma. The anticoagulant therapy with unfractionated heparin was to be continued, targeting an activated partial thromboplastin time (aPTT) at first around 70 s and a week later adjusted to 50–60 s, to balance thrombus stabilization, while minimizing the risk of hematoma. Close monitoring with serial echocardiograms and inflammatory markers was implemented to track the progression of both the thrombus and the hematoma.

Suspecting possible vasculitis in the context of a recent history of suspected DVT and MI, immunoserological tests were performed, all of which were negative except for immunoglobulin G (IgG) class anticardiolipin antibodies. Further investigations are warranted.

### Clinical deterioration and surgical decision

**Figure 3:**
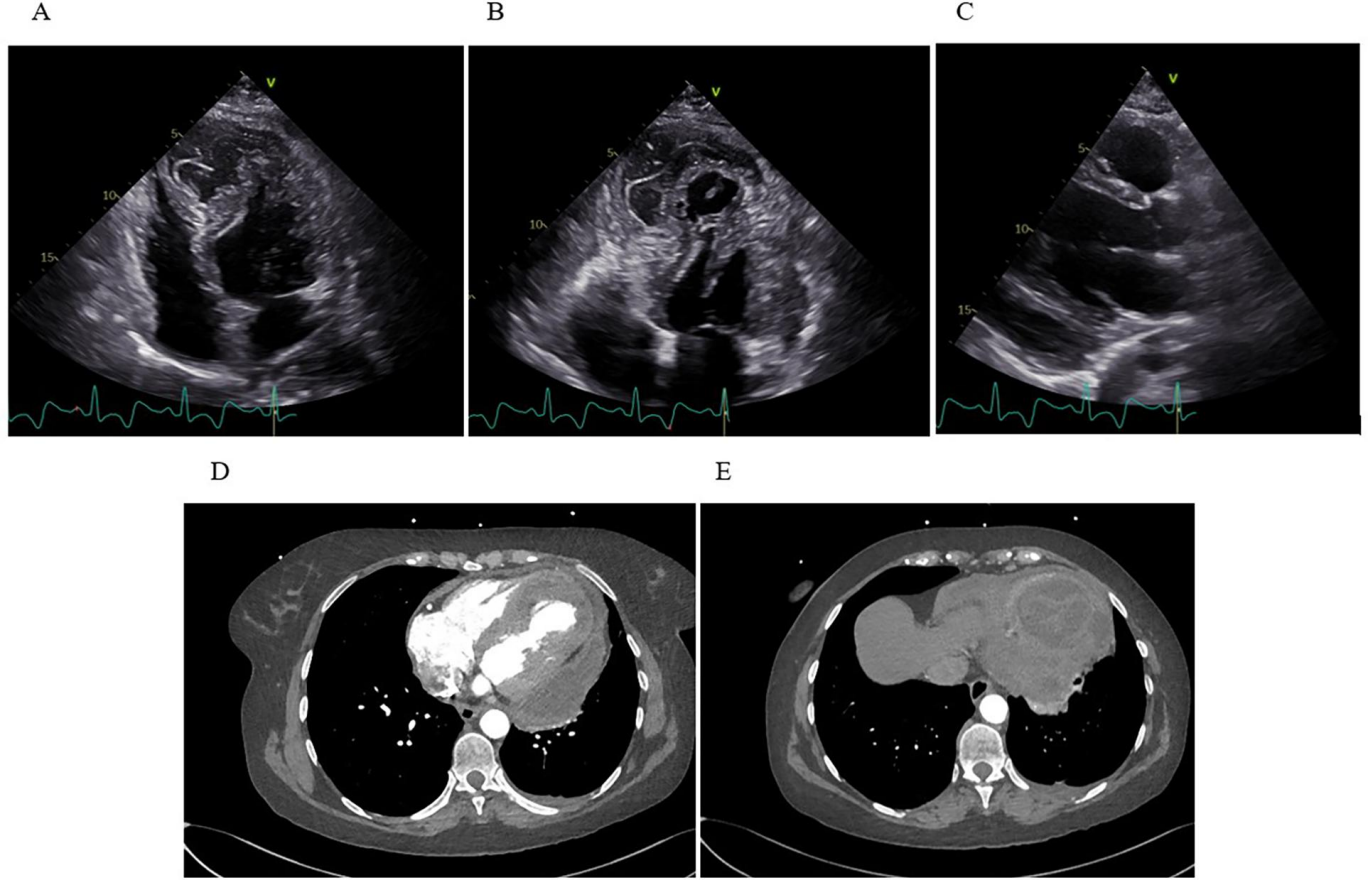
Transthoracic echocardiography and computed tomography. **(A)** Apical four-chamber view showing extensive IDH with marked apical dyskinesia. **(B)** Apical thrombus formation visualized as an echogenic mass at the ventricular apex. **(C)** The parasternal long-axis view reveals a moderate, new pericardial effusion along the inferolateral wall of the LV. **(D)** Axial CT demonstrates a multiloculated, heterogeneous fluid collection within the inferoapical myocardium, with clear communication to a large pericardial effusion along the lateral and posterior walls of the LV. **(E)** A separate axial CT slice shows a clearly defined, round, intramyocardial lesion.

By day 30, the patient again became febrile with rise in inflammatory markers (CRP spiking to 339 mg/L, procalcitonin up to 0.71 µg/L). This prompted a heightened concern for infective hematoma or pericarditis. TTE revealed newly present pericardial effusion along the lateral and inferior wall of the LV with a fluid layer of up to 22 mm. The intramural fluid collection also looked more heterogeneous and potentially communicated with the pericardial space (Figure 3).

Because of a suspected rupture or an expanding dissecting hematoma, an *ad hoc* consultation with cardiovascular surgeons, transplant cardiologists and the CCU cardiology team took place. The plan was to obtain additional imaging to assess the possible communication of the hematoma, evaluate for emergent surgical intervention and complete a “transplant workup” in case the ventricle was too compromised and mechanical support or transplantation were needed post-surgery.

Later that day, the patient underwent additional imaging, including a CT scan of the chest, which showed a large, multi-chambered fluid collection in the inferoapical myocardium, measuring about 65 × 55 × 33 mm, communicating widely through a 20 mm channel with a separate large fluid pocket of approximately 55 × 25 × 100 mm running from the apex along the lateral wall up to the cardiac base (Figure 3). The concern was that the previously noted intramural hematoma had partially liquefied and extended into the pericardial space. The possibility of infection or an abscess could not be fully excluded.

Given the suspicion of infection (or at least an infected/complicated fluid collection), empirical broad-spectrum antibiotics with vancomycin + piperacillin/tazobactam were started the same day. Blood cultures remained negative during this timeframe, but the patient's inflammatory markers were rising.

At a final *ad hoc* meeting, the consensus was that the dissecting, potentially infected inferoapical hematoma necessitated urgent open surgical repair. Surgeons also planned for the possibility of mechanical circulatory support or cardiac transplantation should the ventricle not be salvageable. Preoperatively, unfractionated heparin was discontinued.

**Figure 4:**
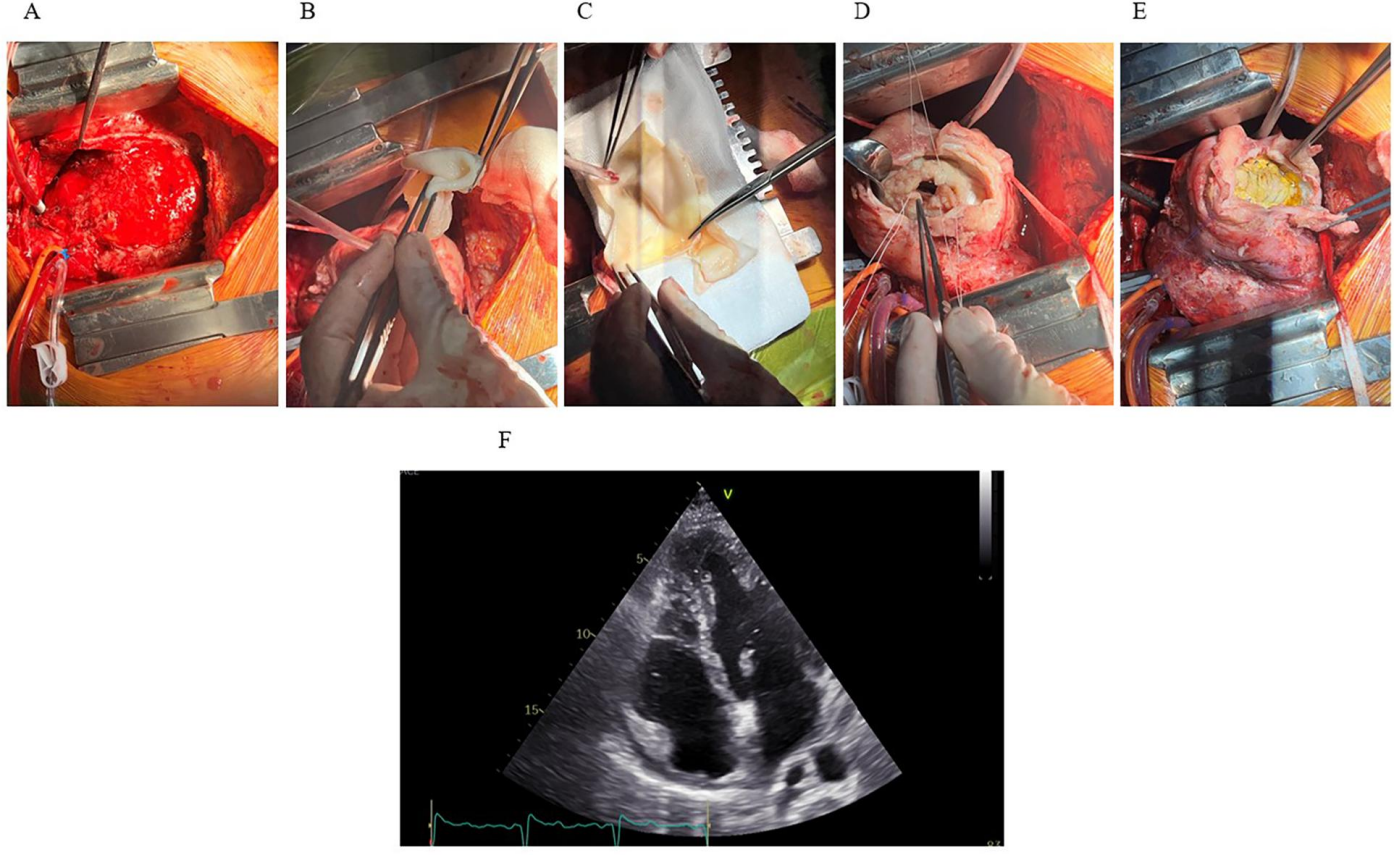
The Dor procedure and outcome. **(A)** Median sternotomy reveals the heart exposed, showing extensive hematoma and inflammation involving the inferoapical myocardial region. **(B,C)** A synthetic patch for reconstruction of the LV geometry. **(D)** LV cavity exposed following resection of infarcted and necrotic myocardium. Sutures placed around the cavity for patch attachment. **(E)** Final positioning and fixation of the synthetic patch. **(F)** Postoperative TTE (apical four-chamber view) demonstrates successful resolution of the IDH and intracavitary thrombus, with only residual apical hypokinesis.

On day 31, the patient was transferred to the operating room, where first the median sternotomy and thorough adhesioloysis of the heart were performed. After sufficient exposure of the left ventricle, the LV aneurysm was opened and an endoventricular circular patch repair (i.e., Dor procedure) of the left ventricle performed using intraoperative tailored bovine pericardial patch (Abbot®, USA) shaped in an elliptical form measuring approximately 5 × 3 centimeters. Due to known ischemic aetiology of the disease no samples of the aneurysmatic tissue was sent for pathology, however, samples of the intramural hematoma were sent for microbiology to exclude the infective component of the disease (Figure 4). The chest was left open for delayed sternal closure and was subsequently closed on the next day once patient's complete hemodynamic stability and adequate haemostasis were established. No additional temporary mechanical circulatory support to achieve patient's hemodynamic stability was needed throughout the perioperative period.

The patient tolerated the procedures well and was extubated on day two, post-operative course was insignificant. A TTE, performed 9 days after surgery revealed good LV systolic function with left-ventricular ejection fraction (LVEF) of 54% and slightly reduced left-ventricular outflow tract (LVOT) velocity-time integral (VTI) of 13 cm (stroke volume 22 mL/m^2^) with hypokinesia of apical septal segment and akinesia of apical inferior segment (Figure 4). Antibiotic treatment was completed on day 8 after surgery with no clinical signs of infection and low inflammatory markers.

On the same day, the patient was discharged home in good general condition, afebrile, and with stable vital signs (blood pressure 96/61 mmHg, sinus rhythm). The sternotomy wound was well-approximated using staples, and the chest tube sites were healing without complication. She was discharged on pantoprazole 40 mg OD, aspirin 100 mg OD, colchicine 0.5 mg BiD, bisoprolol 10 mg OD, empagliflozin 10 mg OD and spironolactone 25 mg OD. We decided against P2Y12 inhibitor in absence of clear acute coronary lesions. ARNI could not be reinitiated due to low blood pressure. A referral was made for post-cardiac surgery rehabilitation. Comprehensive discharge instructions included avoiding strenuous physical activity and adhering to regular follow-ups with her cardiologist to monitor her recovery trajectory.

### Rehabilitation and functional recovery

The patient was enrolled in a structured cardiac rehabilitation program 1.5 months after the procedure, focusing on light aerobic exercises such as walking and stationary cycling, with gradual increases in intensity. Follow-up TTE showed LVEF of 48% with persistent apical hypokinesis but no residual hematoma or pericardial effusion. During her first stress test 1.5 months after the procedure, she achieved a workload of 79 watts [4.6 metabolic equivalents (METs)] without symptoms or complications. Her functional capacity gradually improved, reaching 87 watts (5.0 METs) 2 months after the procedure confirming successful recovery. At discharge from rehabilitation, she was capable of daily activities, advised to continue moderate exercise, and maintain regular follow-ups to ensure continued progress.

## Discussion and conclusions

IDH remains a rare but potentially devastating complication of MI, involving partial or subacute rupture of the infarcted myocardium. leading to the formation of a blood-filled cavity (1, 2). The precise mechanisms underlying its development remain incompletely understood, though it is widely attributed to necrosis-induced weakening of the myocardium and inflammation-driven disruption of the microvasculature. Hypoxia-related endothelial damage and heightened proteolytic activity can further exacerbate this process, particularly under the influence of reperfusion therapy. Additionally, the heart's spiral fiber arrangement may facilitate dissection along planes of least resistance once bleeding has begun (6, 8).

The clinical presentation of IDH is highly variable: some patients demonstrate gradual reabsorption of the hematoma under cautious conservative management, whereas others decompensate rapidly, prompting urgent surgical intervention (5, 8, 10). Hemodynamic compromise, ventricular septal defect, low ejection fraction and ongoing expansion of the lesion are frequently cited as critical indicators that mandate operative repair (4, 6).

In the present case, the patient was initially perceived to have stress-induced cardiomyopathy, but recurrent chest pain, fever, and rising inflammatory markers prompted additional imaging that ultimately demonstrated a large dissecting hematoma localized to the inferoapical wall-an evolution that highlights how dynamic and elusive the clinical course of IDH can be, especially in patients whose early myocardial injury is obscured by nonobstructive coronary findings and probable spontaneous resolution of coronary thrombus.

TTE played a vital role in the early identification of myocardial wall abnormalities, yet it was the use of MRI that provided definitive characterization of myocardial necrosis—confirming the presence of hemorrhage, and identifying a concomitant LV thrombus. MRI is frequently cited as the gold standard for tissue characterization, facilitating the differentiation between true hematoma, pericardial effusions, and alternate causes of cavitation within the LV (4, 8).

Management of IDH remains controversial, largely due to lack of experience with this condition (12). If the lesion is small, localized, and the patient is hemodynamically stable, without risk predictors such as ventricular septal perforation or pericardial effusion, conservative management with close hemodynamic monitoring and careful echocardiographic follow-up is justified, as spontaneous reabsorption of the hematoma can occur (3, 4, 8). However, our patient displayed progressive dissection into the pericardial space, an enlarging intracavitary thrombus, and eventually signs of systemic inflammation with rising inflammatory markers and febrile episodes, all of which compounded the complexity of her treatment. These factors made a purely conservative strategy untenable and forced a transition to urgent surgical intervention.

A particularly challenging aspect of this case was the concurrent need for anticoagulation in the presence of both an intramural hematoma and an LV thrombus. This dual pathology raised concern for systemic embolization, necessitating anticoagulation, however, the simultaneous presence of the intramyocardial hematoma demanded caution, as anticoagulation might exacerbate the dissection and potentially lead to bleeding into the pericardial space (11, 13). To balance these risks, a stepwise approach to anticoagulation was employed-using a continuous unfractionated heparin infusion, given that the immediate threat from the expanding thrombus was deemed greater than that from the hematoma-coupled with rigorous serial imaging to monitor for any signs of hematoma progression.

A further complicating factor was the patient's persistent fever, escalating inflammatory markers and pericardial effusion which raised suspicions of hematoma infection or infected pericardial fluid. Although published data on overt sepsis within an intramyocardial dissection are scarce, any potential infection could further compromise already weakened myocardial layers. In such cases, some clinicians may consider urgent surgical drainage and debridement, as well as broad-spectrum antibiotic therapy, to reduce the likelihood of abscess formation. In this scenario, empirical broad-spectrum antibiotics were promptly administered to cover possible bacterial contamination of the dissected cavity. Although blood cultures remained negative, this patient's clinical deterioration and imaging findings illustrated how infection risk may tip the balance toward urgent surgical intervention.

Surgery remains the definitive option once evidence of compromised hemodynamics or impending rupture emerges: specific tailored repairs enable resection of necrotic myocardium and containment of the hematoma using an endoventricular patch, effectively restoring geometry and reducing the propensity for rebleeding (8, 11, 12).

In this case, the Dor procedure-resecting devitalized myocardium, reconstructing the LV with an endoventricular patch, and closing the dissected cavity-provided a definitive solution by stabilizing the ventricle and preventing further rupture. Postoperative resolution of the hematoma and thrombus, along with significant improvement in LVEF, highlights the capacity for functional myocardial recovery once the dissecting cavity is controlled, the load on viable myocardium is reduced and mechanical integrity is restored.

Following a technically challenging operation, the patient's rehabilitation phase aimed at gradually restoring her cardiovascular fitness. Postoperative TTE confirmed improvement in LVEF alongside diminishing inflammatory markers, providing confidence in the surgical repair's effectiveness. Long-term vigilance with scheduled echocardiographic evaluations is advisable, considering the potential risk of residual or recurrent fluid collections, subtle re-dissection, and possible new thrombotic events (3, 5, 14).

Overall, the present scenario highlights how IDH invariably demands multidisciplinary collaboration, frequent imaging to adapt therapy in real time, judicious balancing of anticoagulant needs, and readiness to escalate to surgical repair when the hematoma threatens free-wall integrity. Further studies are warranted to refine diagnostic algorithms and establish evidence-based management protocols.

## Data Availability

The original contributions presented in the study are included in the article/Supplementary Material, further inquiries can be directed to the corresponding author.
