# Supplementary material for: Surgical treatment of intramyocardial dissecting hematoma—a case report and literature review
Source: Front Cardiovasc Med. 2025 Dec 1;12:1700770. doi: 10.3389/fcvm.2025.1700770 (PMC12702927; doi:10.3389/fcvm.2025.1700770)
Supplement: Supplementary file 1 [file Datasheet1.pdf]

Supplementary Table 1. Patient timeline.

Timeline	
**Date**	**Clinical/Diagnostic Event**
**11 Sep 2024**	Patient presents with pressure-like chest pain, mild dyspnoea, suspicious for anterior STEMI.
**12 Sep**	Coronary angiography shows no significant stenosis; ventriculography suggests Takotsubo. Managed conservatively.
**14–15 Sep**	Patient experiences fever; streptococcal throat infection diagnosed. CRP elevated (192 mg/L). Troponin rises again (up to 4584 ng/L).
**16 Sep**	TTE: suspicious “layering” in LV wall. CT: large inferoapical transmural infarction with signs of haemorrhage into the necrotic myocardial wall. Transferred to tertiary center.
**20 Sep**	Cardiac MRI confirms large IDH (60 × 55 × 30 mm), apical thrombus and a mild pericardial effusion. Low-intensity heparin infusion started.
**Late Sep–Oct**	Imaging shows slowly enlarging haematoma and a growing apical thrombus; CRP fluctuates. Adjusted anticoagulation approach; intensive monitoring.
**10–11 Oct**	Patient becomes febrile; CT: large, possibly infected, multi-chambered fluid collection communicating with the pericardial space. Broad-spectrum antibiotics started.
**12 Oct**	Urgent surgical repair (Dor procedure) for suspected infected dissecting haematoma. Successful operation and stable postoperative course.
**22 Oct**	Patient discharged home with improving LVEF 54%, no residual haematoma, no pericardial effusion.
**Late Nov–Dec**	Ongoing cardiac rehabilitation. Follow-up TTE: LVEF 48%, no residual haematoma, improved functional capacity.

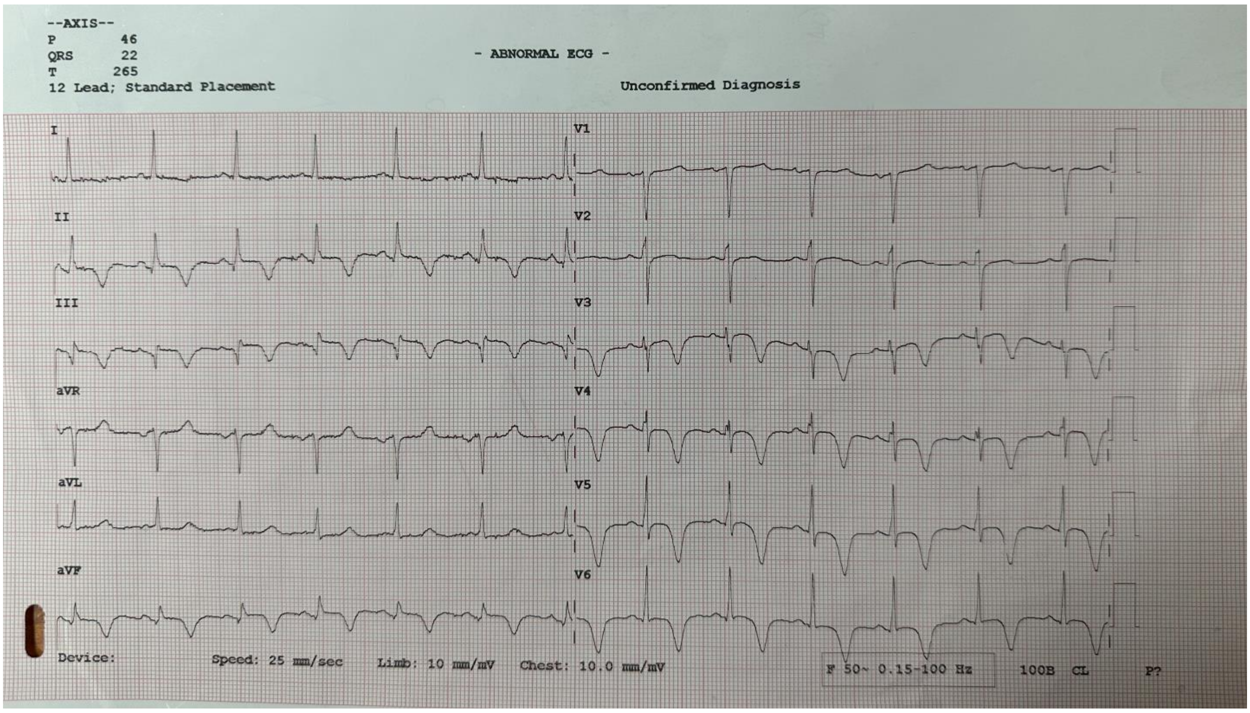

Supplementary Figure 1: Initial ECG demonstrating sinus rhythm with T-wave inversions in leads II, III, aVF, and precordial leads V3–V6, suggesting acute myocardial ischemia.

A B

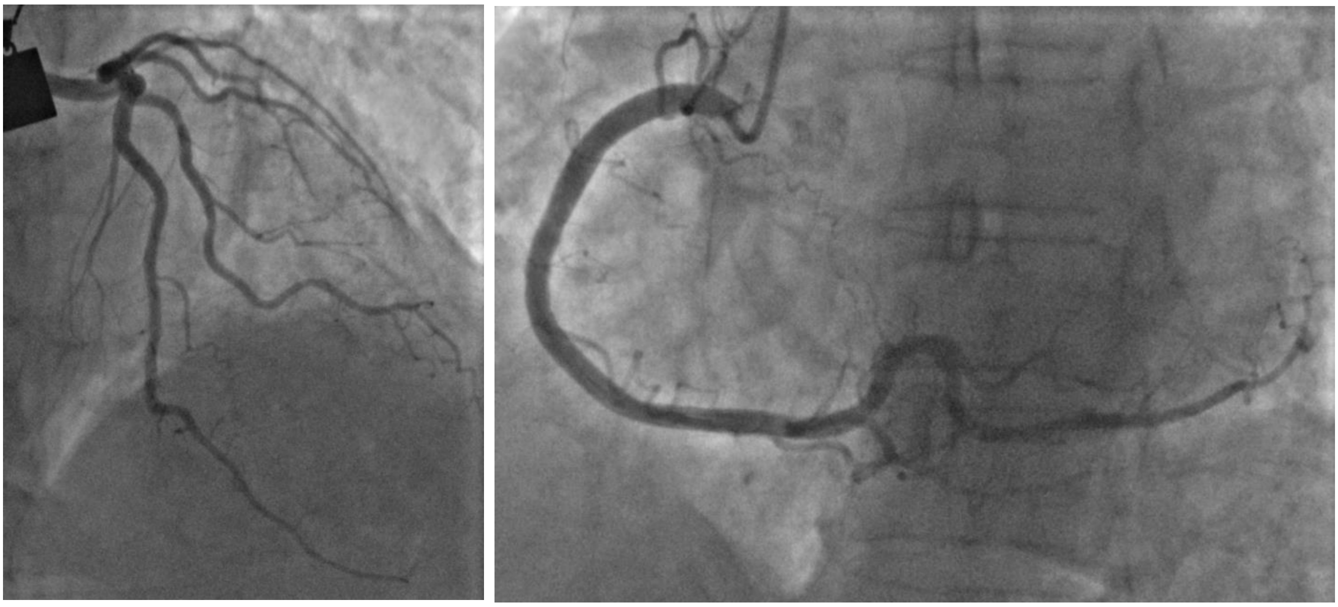

Supplementary Figure 2: (A) Left coronary angiography showing normal flow in the left anterior descending artery and left circumflex artery without significant obstruction. **(B)** Right coronary angiography showing a normal right coronary artery with no significant stenosis.

A B

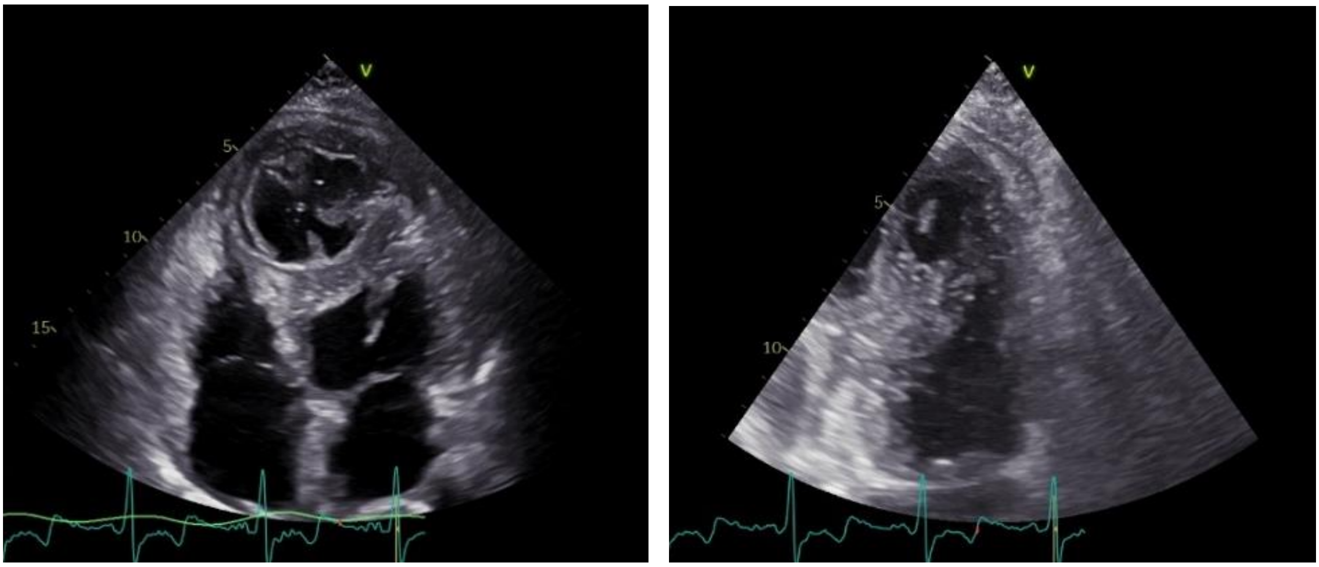

Supplementary Figure 3: Transthoracic echocardiography **(A)** Apical four-chamber view revealing enlargement and increased heterogeneity of the intramyocardial dissecting hematoma. **(B)** Apical two-chamber view demonstrates a persistent intracavitary thrombus
